# Lower psoas mass indicates worse prognosis in percutaneous vertebroplasty-treated osteoporotic vertebral compression fracture

**DOI:** 10.1038/s41598-024-64626-z

**Published:** 2024-06-16

**Authors:** Kai Sun, Jianjun Liu, Haoran Zhu, Jiajia Wang, Haiwu Wan, Bo Huang, Qinglin Zhang, Guoliang Chen

**Affiliations:** 1 Department of Orthopedic Surgery, Jiujiang University Affiliated Hospital, Jiujiang, 332006 China; 2 Department of Orthopedic Surgery, The Fifth Affiliated Hospital of Jinan University (Heyuan Shenhe People’s Hospital), Heyuan, 517000 China; 3 Department of Orthopedic Surgery, Dongguan Qiaotou Hospital, Dongguan, 523539 China; 4 Department of Orthopedic Surgery, The First Affiliated Hospital of Jinan University, Guangzhou, 510632 China

**Keywords:** Psoas mass, Elderly, Osteoporotic vertebral compression fractures, Percutaneous vertebroplasty, Prognosis, Diseases, Medical research

## Abstract

The correlation between lower psoas mass and the prognosis of osteoporotic vertebral compression fractures (OVCF) is still unclear. This study aims to investigate the impact of lower psoas mass on the prognosis of patients undergoing percutaneous vertebroplasty (PVP). One hundred and sixty-three elderly patients who underwent single-segment PVP from January 2018 to December 2021 were included. The psoas to L4 vertebral index (PLVI) via MRI were measured to assess psoas mass. Patients were divided into high PLVI (> 0.79) and low PLVI (≤ 0.79) groups based on the median PLVI in the cohort. The basic information (age, gender, body mass index (BMI) and bone mineral density (BMD)), surgical intervention-related elements (duration of operation, latency to ambulation, period of hospital stay, and surgical site), postoperative clinical outcomes (Visual Analog Scale (VAS) scores, Oswestry Disability Index (ODI) scores, Japanese Orthopaedic Association (JOA) scores), and incidence of secondary fractures) were compared. Patients showed no statistically significant differences in terms of age, gender, surgical sute, BMI, BMD and preoperative VAS, ODI, JOA scores (*P* > 0.05) between the two groups. However, there were significant differences in terms of latency to ambulation, period of hospital stay (*P* < 0.05). VAS, ODI, and JOA scores at 1, 6, and 12 months after surgery showed that the high PLVI group had significantly better outcomes than the low PLVI group (*P* < 0.05). Additionally, the low PLVI group had a significantly higher incidence of recurrent fracture (*P* < 0.05). Lower psoas mass can reduce the clinical effect of PVP in patients with osteoporotic vertebral compression fractures, and is a risk factor for recurrent vertebral fracture.

## Introduction

Oteoporotic fracture is becoming increasingly common due to the aging society, and vertebral compression fractures caused by osteoporosis have become one of the most significant global health issues in the elderly^1^. Approximately 20% of the global population is over 70 years old, and 16% of postmenopausal women have experienced osteoporotic vertebral compression fractures (OVCF)^2^. Studies have shown that the incidence of OVCF in patients over 50 years old in China has increased by 1.79 times, from 85.21 per 100,000 person-year in 2013 to 152.13 per 100,000 person-years in 2017^3^. Previously, conservative treatment was the major approach for OVCF, but long-term bed rest resulted in nursing difficulties, persistent pain, kyphosis, weight loss, depression, decreased quality of life, and even death, leading to a significant socio-economic burden^4^. Percutaneous vertebroplasty (PVP) is currently the most common surgical treatment for OVCF. This method is minimally invasive, time-efficient, and provides rapid symptom relief^5^. In a study involving 229 patients with OVCF who underwent PVP, all patients achieved good pain relief after 1 year of follow-up^6^. However, several studies have reported that some patients may experience residual back pain and recurrent vertebral fractures after PVP^7,8^. Previous study has indicated that pain is related to patient's bone mineral density (BMD), cement leakage, volume of cement injection, postoperative vertebral infection, recurrent fractures, and loosening of the cement-bone interface^9^. In recent years, an increasing number of studies have suggested that sarcopenia is a risk factor that affects the efficacy of PVP^10^. There is a clear definition and diagnostic criteria for sarcopenia, which includes indicators such as muscle strength, muscle quantity, and physical performance^11–13^. In patients with OVCF, assessing muscle strength becomes challenging due to their limited mobility, rendering previous measurement techniques less effective. Recent research suggests that using the total cross-sectional area(CSA) of the lumbar muscles as an alternative indicator for diagnosing sarcopenia is feasible^14,15^.Given that the cut-off value of CSA may vary with body size, it needs to be adjusted according to body size. But the lack of muscle strength measurement has led to much controversy.Given that the cut-off value of CSA may vary with body size, it needs to be adjusted according to body size.Therefore, we adopted the PLVI assess mass of psoas,for this method provides standardized values based on individual patient's body habitus^16^. But its effect on the surgical outcomes of OVCF in the elderly has not been reported. This study aims to explore the impact of lower psoas mass on the postoperative effects of PVP and provide a theoretical basis for preventing post-PVP vertebral fractures in clinical practice.

## Methods

This is a retrospective cohort study. Patients with OVCF and were treated by PVP in our department between January 2018 and December 2021 were enrolled. This study was approved by the Institutional Review Board of the studied hospitals. The clinical methodologies were in strict accordance with the tenets of the Declaration of Helsinki. One hundred and sixty-three elderly patients (age range: 60–91 years) with single-level OVCF who underwent PVP treatment were included. Patients with history of spinal surgery, diseases such as Kummel disease, spinal deformities, or spinal tumors, diseases affecting bone metabolism (e.g., chronic renal failure, hyperparathyroidism) were excluded. Finally, 144 patients were enrolled to compare. The independent variables encompassed basic information (age, gender, body mass index (BMI) and BMD), elements related to surgical intervention (duration of operation, latency to ambulation, period of hospital stay, and surgical site), alongside postoperative clinical indices (Visual Analog Scale (VAS) scores, Oswestry Disability Index (ODI) scores and Japanese Orthopaedic Association (JOA) scores).

### Ethics approval

This research protocol was approved by the Institutional Review Board of Jiujiang University Affiliated Hospital and The First Affiliated Hospital of Jinan University. The clinical procedures adhered to the principles of the declaration of Helsinki.

### Consent to participate

Informed consent was obtained from all individual participants included in the study.

## Radiological assessment

**Figure 1:**
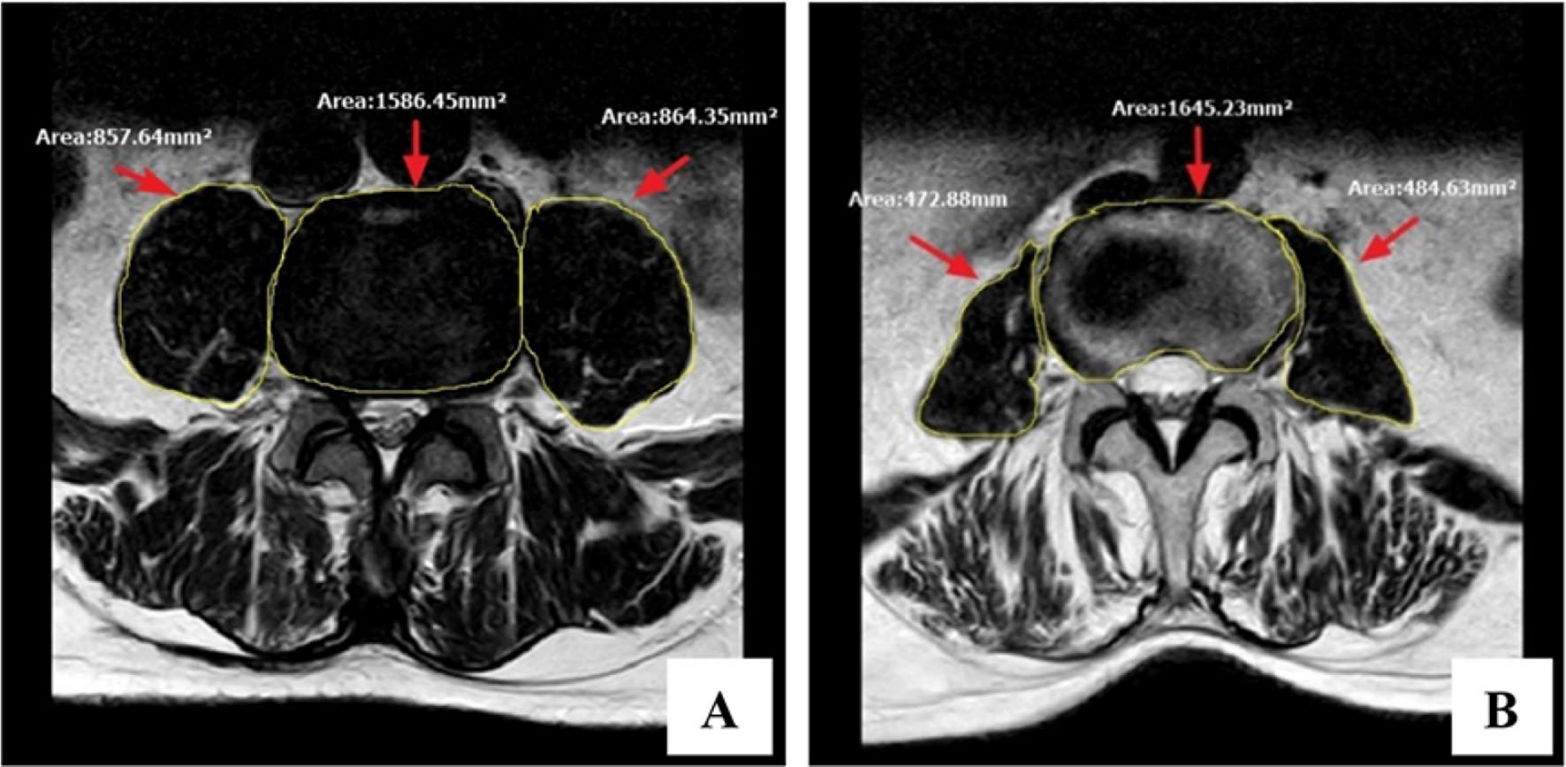
The calculation of PLVI. PLVI = [right PCSA + left PCSA]/ L4 vertebral CSA. Left: the representative case of High PLVI, right: the representative case of Low PLVI. PLVI indicates psoas to L4 vertebral index, CSA indicates cross-sectional area, PCSA indicates psoas CSA.

All measurements were performed by the same two researchers who received professional training independently and repeated three times. MRI scans were conducted using the same protocol for all patients, with measurements taken at the level below the upper endplate of L4. The contours of the left and right psoas muscles and vertebrae were manually outlined on T2-weighted axial images using the Picture Archiving and Communication System (PACS) of the hospital. Image J software (U.S. National Institutes of Health, Bethesda, MD, USA) was used to evaluate the MR images to obtain the psoas to L4 vertebral index (PLVI), which represents the ratio of the bilateral psoas cross-sectional area (PCSA) at the level below the upper endplate of L4 to the vertebral body. The PLVI value at the 50th percentile was determined, and patients were classified into two groups based on their relationship to the median of the cohort: high PLVI (> 0.79) and low PLVI (≤ 0.79). (Fig. 1).

## Surgical procedure

Percutaneous vertebroplasty (PVP) was consistently executed by the designated medical team. In brief, the patient was positioned prone on the operating table. Following standard aseptic protocol, the procedural area was disinfected, draped, and locally anesthetized. A 4-mm skin incision was then precisely created at the intended site overlying the facet joints on either side of the afflicted vertebral segment. Utilizing a specialized 2.5-mm diameter puncture needle, the operator navigated through the vertebral pedicle to access the core of the fractured vertebral body. Upon establishing a viable channel, a controlled volume of 3–5 ml of polymethylmethacrylate bone cement was incrementally injected into the vertebral body. Verification of the accurate placement and containment of the cement within the vertebral body was ascertained under the vigilant guidance of C-arm fluoroscopy. Following a requisite 8-min interlude for cement hardening, the puncture needle was withdrawn. The procedure culminated with the suture of the incision, followed by the application of sterile dressings. Postoperative anti-osteoporosis treatment includes the use of zoledronic acid for each patient during hospitalization, as well as daily foundational supplements of calcium and vitamin D upon discharge.

## Statistical analysis

Data underwent statistical evaluation using SPSS 24.0 (IBM Corp., Armonk, NY, USA), with all values delineated as means ± standard deviation (SD). The study’s focal point was the incidence rate of successive osteoporotic vertebral fractures, designated as the dependent variable. The independent variables encompassed demographic factors (age, gender, BMI, BMD), elements related to surgical intervention (duration of operation, latency to ambulation, period of hospital stay, and surgical site), alongside postoperative clinical indices (VAS, ODI, and JOA scores). Univariate analyses were performed employing Fisher's exact test for categorical data, and the Wilcoxon signed-rank test for continuous data, to elucidate differences between cohorts. A threshold of *P* < 0.05 was established for statistical significance.

## Results

In this study, we encompassed a cohort of 144 patients with OVCF who fulfilled our inclusion criteria from a larger group of 163 individuals. The cohort was dichotomized into two groups: one with a higher PLVI comprising 72 patients, and another with a lower PLVI, also consisting of 72 patients. Comparative analysis revealed no statistically significant disparities in surgical segments, age, gender, operative duration, BMI, or BMD between the groups (Table 1). Nonetheless, a statistically significant acceleration in the time to ambulation and reduced hospital stay was noted in the high PLVI group (*P* < 0.05), indicating a quicker resumption of daily activities for these patients (Table 2).

**Table 1:** Surgical segmental division in two groups.

	High PLVI group	Low PLVI group
T9	3	4
T10	5	3
T11	12	15
T12	15	13
L1	19	18
L2	12	15
L3	6	5
Total	72	72

**Table 2:** Comparison of baseline data between the two groups.

Item	Age at surgery (year)	Gender (M/F)	Operation time (min)	BMI	BMD	Latency to ambulation (hour)	Hospitalization time (hour)
High PLVI n = 72	72.6 ± 8.2	23(M)/49(F)	32.6 ± 12.2	22.6 ± 2.7	− 3.4 ± 0.4	25.6 ± 10.7	112.8 ± 12.5
Low PLVI n = 72	75 ± 10.8	19(M)/53(F)	30.3 ± 12.8	21.2 ± 1.9	− 3.7 ± 0.6	64.2 ± 13.9	166.9 ± 11.1
*P* value	0.901	0.870	0.881	0.910	0.890	< 0.001	0.010

BMI indicates body mass index, BMD indicates bone mineral density, PLVI indicates psoas to L4 vertebral index.

Preoperative assessments of pain and functional status, measured via the VAS, ODI and JOA scores, did not differ significantly between the two groups (*P* > 0.05). However, at subsequent follow-ups post-surgery, the high PLVI group exhibited superior outcomes in VAS, ODI, and JOA scores (*P* < 0.05), corroborating a potential trend towards enhanced pain management and functional recovery in this subgroup (Table 3).

**Table 3:** Comparisons of clinical outcomes between the two groups.

Group	Preoperative	1 month postoperative	2-month postoperative	12-month postoperative
VAS score				
High PLVI	7.3 ± 0.8	1.7 ± 0.3	1.2 ± 0.7	0.3 ± 0.4
Low PLVI	7.4 ± 0.4	3.1 ± 0.5	2.0 ± 0.5	0.9 ± 0.2
*P* value	0.960	< 0.001	0.004	0.010
ODI				
High PLVI	64.4 ± 7.3	36.5 ± 4.9	24.7 ± 7.2	19.3 ± 6.2
Low PLVI	63.8 ± 4.6	44.6 ± 5.7	29.5 ± 6.2	21.8 ± 4.8
*P* value	0.950	0.003	0.004	0.021
JOA score				
High PLVI	10.5 ± 5.9	20.7 ± 5.1	23.4 ± 4.8	25.1 ± 3.1
Low PLVI	11.0 ± 4.6	17.2 ± 3.5	21.4 ± 5.2	23.1 ± 4.5
*P* value	0.930	0.021	0.034	0.020

VAS indicates Visual Analog Scale, ODI indicates Oswestry Disability Index and JOA indicates Japanese Orthopaedic Association.

## Complications

**Figure 2:**
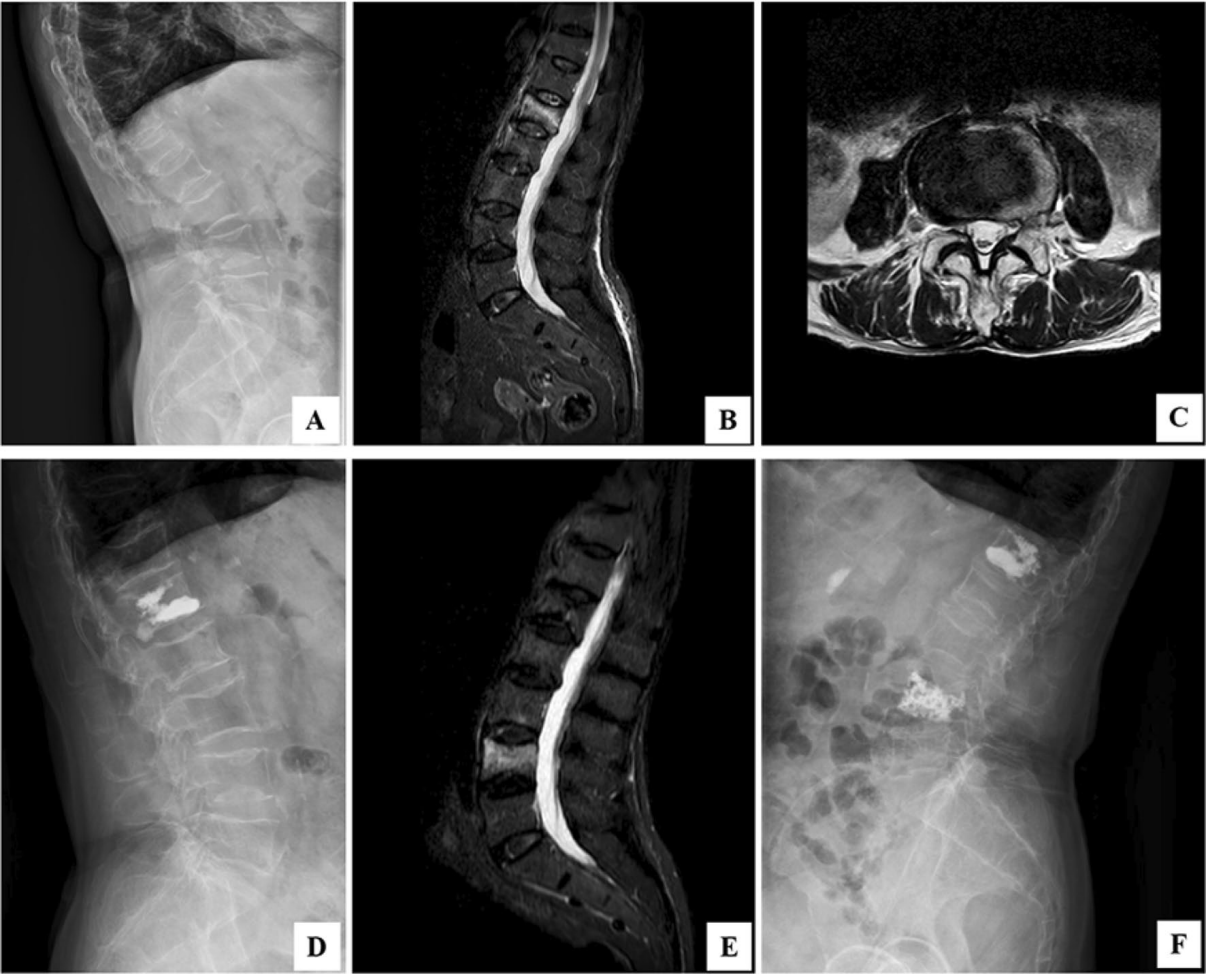
Typical re-fracture case of central sarcopenia, a 72-year-old female patient. On April 25, 2019, fall caused back pain, and then DR (**A**), MR (**B**, **C**) were performed. After admission, PVP was performed, and DR Was reexamined after operation (**D**). Five months later, low back pain recurred, and a reexamination of MR revealed a L4 fracture (**E**). The patient was readmitted for PVP (**F**).

Within the cohort exhibiting a high percentage of lumbar vertebral involvement, a mere 2.78% (2 cases) reported persistent low back pain (LBP) at the final follow-up. In contrast, the group with a lower PLVI presented with 8.33% (6 cases) experiencing residual LBP. This disparity is statistically significant (*P* < 0.05). Alleviation of symptoms was partially attained through pharmacological intervention with anti-osteoporotic medication, complemented by physical therapy and structured guidance for lumbar muscle strengthening exercises. Over the span of one year, the incidence of subsequent osteoporotic vertebral fractures (SOVF) was observed to be 4.16% (3 new cases) in the high PLVI group, as opposed to 11.1% (8 cases) in the low PLVI group. The statistical significance of this difference (*P* < 0.05) suggests a noteworthy correlation between lower psoas mass and the occurrence of SOVF (Fig. 2).

## Discussion

The incidence of sarcopenia is notably elevated among elderly individuals, particularly accentuated within Asian demographics^17^.Some scholars use PLVI to represent central sarcopenia. Clinical evidence substantiates the correlation between PLVI and the prognosis of various geriatric ailments, thus garnering increasing favor among scholars^18^. Previous studies have shown a clear association between PLVI and adverse outcomes following liver transplantation and open repair of ruptured aortic aneurysm^19,20^. Our research discovered a correlation between PLVI and the surgical outcomes of OVCF patients, highlighting the potential of lower psoas mass as a prognostic indicator for elderly patients. The vast majority of OVCF patients undergo lumbar spine MRI preoperatively, providing measurement conditions early on. Additionally, assessing radiographic results in the early stages of patient hospitalization minimizes interference from other injuries or treatments, thus better reflecting the patient's baseline status. Through follow-up of cases within our group, we observed a correlation between lower psoas mass and OVCF. Osteoporosis and falls are predominant contributors to vertebral compression fractures among the elderly-falls represent external causative factors, while osteoporosis is an internal one. Contemporary research indicates a substantial interrelationship between sarcopenia and osteoporosis, which reciprocally exacerbate one another, culminating in detrimental consequences such as falls and fractures^21^. Sarcopenia is widely acknowledged as an independent risk factor for OVCF, impacting the prognosis distinct from osteoporosis^22^. Contrarily, some academics contend that sarcopenia, through its intimate association with osteoporosis, enhances the likelihood of OVCF, though it is not an independent risk factor^23^. Furthermore, falls is a significant risk factor for fragility fractures in the elderly, encompassing OVCF. Our study found that the two patient groups had similar levels of osteoporosis. Therefore, for the occurrence of re-fracture, the increased risk may be attributed more to lower psoas mass causing falls. We believe that lower psoas mass increases the risk of falls, consequently elevating the risk of fractures. Existing studies corroborate that lower PLVI elevates the risk of falls, thereby amplifying the risk of fractures^24^. An extensive study spanning 13,101 participants across five nations revealed that individuals with sarcopenia experienced a markedly elevated rate of fall-induced injuries and a 1.85-fold increase in fracture incidence due to falls when contrasted with non-sarcopenic counterparts^25^. Whether intensifying osteoporosis or augmenting the propensity for falls, sarcopenia invariably precipitates OVCF, the decline of physical function in the elderly, and an escalated risk of fractures. A separate investigation involving 120 patients, which assessed sarcopenia by measuring the lumbar muscle area, determined that a diminished area subsequent to muscle fat infiltration constituted an independent risk factor for recurrent fractures PVP surgery, irrespective of bone density. This correlation may stem from impaired balance due to muscle fat infiltration, thereby heightening the risk of recurrent fractures^26,27^. Furthermore, current studies propose that paravertebral muscles are vital in upholding spinal stability, as they inhibit excessive flexion and mitigate vertebral load. In the absence of muscular support, the spine's threshold for compression prior to flexion is a mere 2 kilograms^28^. Individuals with sarcopenia are bereft of this muscular defense, potentially precipitating repeated spinal compression fractures^29^.

The debate continues over sarcopenia's influence on the postoperative prognosis in OVCF patients. Research encompassing 101 subjects indicates that muscle atrophy may compromise the clinical efficacy of PVP interventions^30^. Nevertheless, this assertion is not universally supported. An investigation involving 116 individuals receiving percutaneous kyphoplasty for vertebral fractures discerned no notable disparity in postoperative results between patients with and without sarcopenia^31^. Contrarily, additional research highlighted that sarcopenic patients experienced a significantly greater degree of persistent lower back pain following surgery, possibly attributed to minor micro-movements within the vertebrae subsequent to PVP^32^. In instances where lower back muscles are robust and potent, they can sustain spinal equilibrium. In contrast, muscles enfeebled by fatty degeneration fail to preserve vertebral stability, culminating in persistent lower back discomfort^33,34^. Bayram et al. disclosed that within a cohort of 103 OVCF patients undergoing PVP, those with sarcopenia (PVLI less than 0.603) faced a markedly increased mortality rate in comparison to their counterparts with a higher PVLI (43.1% vs 0%, *P* = 0.001)^35^. Such revelations underscore the clinically substantial ramifications of sarcopenia on the postoperative trajectory of OVCF patients treated with PVP.

This study pioneers the assessment of the correlation between psoas mass, as measured by the PVLI, and postoperative outcomes in patients undergoing PVP. The findings reveal notable deficiencies in lower psoas mass patients, both regarding alleviation of postoperative pain and the frequency of subsequent fractures. These observations are in harmony with the majority of existing literature on the subject. In light of these insights, it is recommended that patients presenting with lower psoas mass receive thorough evaluations in the domains of rehabilitation medicine and nutrition during the perioperative phase. The focus should be on optimizing nutritional status and fortifying muscle integrity. Furthermore, in the postoperative rehabilitation phase, there should be an emphasis on lumbar muscle exercises to augment muscle strength and mitigate muscle atrophy. Despite our findings, this study has several limitations. Given our relatively small sample size, we aim to gather more case data in the future. The median PLVI cutoff of 0.79, which led us to divide the cohort into "high" and "low" PLVI groups, may not be effective in other populations. Further collaboration with larger cohorts may help clarify this issue. Additionally, parameters such as sagittal balance and the efficacy of osteoporosis treatment were not included in our subsequent evaluations, which represents another avenue for future research.

## Conclusion

Our investigation utilized MR imaging to measure psoas mass through the PVLI, affirming its deleterious effects on postoperative functional recuperation and the heightened likelihood of recurrent fractures in patients with OVCF treated with PVP. We advocate for the standard application of this imaging modality in the preoperative evaluation of OVCF sufferers to diminish the rate of recurring fractures and curtail postoperative complications via proactive interventions.

## Data Availability

The data that support the findings of this study are not openly available due to reasons of sensitivity and are available from the corresponding author upon reasonable request.

## References

[1] Mo X, Zhao S, Wen Z (2021). High prevalence of osteoporosis in patients undergoing spine surgery in China. BMC Geriatr..

[2] Karmakar A, Acharya S, Biswas D, Sau A (2017). Evaluation of percutaneous vertebroplasty for management of symptomatic osteoporotic compression fracture. J. Clin. Diagn. Res..

[3] Shen L, Luo K, Deng X, Liu J (2023). A commentary on 'Incidence and cost of vertebral fracture in urban China: A five-year population-based cohort study'. Int. J. Surg..

[4] Center JR, Nguyen TV, Schneider D, Sambrook PN, Eisman JA (1999). Mortality after all major types of osteoporotic fracture in men and women: An observational study. Lancet.

[5] Liu Z, Zhang X, Liu H, Wang D (2022). A nomogram for short-term recurrent pain after percutaneous vertebroplasty for osteoporotic vertebral compression fractures. Osteoporos. Int..

[6] Klazen CA, Lohle PN, de Vries J (2010). Vertebroplasty versus conservative treatment in acute osteoporotic vertebral compression fractures (Vertos II): An open-label randomised trial. Lancet.

[7] Li Y, Yue J, Huang M (2020). Risk factors for postoperative residual back pain after percutaneous kyphoplasty for osteoporotic vertebral compression fractures. Eur. Spine J..

[8] Hwang SH, Cho PG, Kim KT, Kim KN, Kim SH, Noh SH (2023). What are the risk factors for a second osteoporotic vertebral compression fracture. Spine J..

[9] McCarthy J, Davis A (2016). Diagnosis and management of vertebral compression fractures. Am. Fam. Physician.

[10] Ninomiya G (2017). Clinical impact of sarcopenia on prognosis in pancreatic ductal adenocarcinoma: A retrospective cohort study. Int. J. Surg..

[11] Chen LK, Woo J, Assantachai P (2020). Asian Working Group for Sarcopenia: 2019 Consensus update on sarcopenia diagnosis and treatment. J. Am. Med. Dir. Assoc..

[12] Cruz-Jentoft AJ, Baeyens JP, Bauer JM (2010). Sarcopenia: European consensus on definition and diagnosis: Report of the European Working Group on Sarcopenia in Older People. Age Ageing.

[13] Cruz-Jentoft AJ, Bahat G, Bauer J (2019). Sarcopenia: Revised European consensus on definition and diagnosis. Age Ageing.

[14] Sanchez-Rodriguez D, Marco E, Cruz-Jentoft AJ (2020). Defining sarcopenia: Some caveats and challenges. Curr. Opin. Clin. Nutr. Metab. Care.

[15] Cooper C, Dere W, Evans W (2012). Frailty and sarcopenia: Definitions and outcome parameters. Osteoporos. Int..

[16] Ebbeling L, Grabo DJ, Shashaty M (2014). Psoas:lumbar vertebra index: Central sarcopenia independently predicts morbidity in elderly trauma patients. Eur. J. Trauma Emerg. Surg..

[17] Papadopoulou SK (2020). Sarcopenia: A contemporary health problem among older adult populations. Nutrients.

[18] Albano D, Messina C, Vitale J, Sconfienza LM (2020). Imaging of sarcopenia: Old evidence and new insights. Eur. Radiol..

[19] Leunis S, Vandecruys M, Van Craenenbroeck AH (2023). Sarcopenia in end-stage liver disease and after liver transplantation. Acta Gastroenterol. Belg..

[20] Lee JS, He K, Harbaugh CM (2011). Frailty, core muscle size, and mortality in patients undergoing open abdominal aortic aneurysm repair. J. Vasc. Surg..

[21] Jeon I, Kim SW, Yu D (2022). Paraspinal muscle fatty degeneration as a predictor of progressive vertebral collapse in osteoporotic vertebral compression fractures. Spine J..

[22] Lidar S, Salame K, Chua M (2022). Sarcopenia is an independent risk factor for subsequent osteoporotic vertebral fractures following percutaneous cement augmentation in elderly patients. J. Clin. Med..

[23] Anand, A., Shetty, A. P., Renjith, K. R., K S S, Kanna, R. M., & Rajasekaran, S. Does sarcopenia increase the risk for fresh vertebral fragility fractures?: a case-control study. *Asian Spine J*. 14(1): 17–24 (2020). 10.31616/asj.2019.0049 PMC7010510 31575110

[24] Schneider DA, Trence DL (2019). Possible role of nutrition in prevention of sarcopenia and falls. Endocr. Pract..

[25] Yeung S, Reijnierse EM, Pham VK (2019). Sarcopenia and its association with falls and fractures in older adults: A systematic review and meta-analysis. J. Cachexia Sarcopenia Muscle.

[26] Pfeifer M, Sinaki M, Geusens P, Boonen S, Preisinger E, Minne HW (2004). Musculoskeletal rehabilitation in osteoporosis: A review. J. Bone Miner. Res..

[27] Lee DG, Bae JH (2023). Fatty infiltration of the multifidus muscle independently increases osteoporotic vertebral compression fracture risk. BMC Musculoskelet. Disord..

[28] Nachemson A (1966). The load on lumbar disks in different positions of the body. Clin. Orthop. Relat. Res..

[29] Takahashi S, Hoshino M, Takayama K (2020). The natural course of the paravertebral muscles after the onset of osteoporotic vertebral fracture. Osteoporos. Int..

[30] Peng Y, Wu X, Ma X, Xu D, Wang Y, Xia D (2023). Comparison between the clinical effect of percutaneous kyphoplasty for osteoporosis vertebral compression fracture patient with or without sarcopenia: A retrospective cohort study. Int. J. Gen. Med..

[31] Ohyama S, Hoshino M, Takahashi S (2021). Presence of sarcopenia does not affect the clinical results of balloon kyphoplasty for acute osteoporotic vertebral fracture. Sci. Rep..

[32] Lu W, Teng Z, Chen J (2023). A pain that is easily overlooked: Referred pain caused by OVCF. J. Pain Res..

[33] Wang W, Sun Z, Li W, Chen Z (2020). The effect of paraspinal muscle on functional status and recovery in patients with lumbar spinal stenosis. J. Orthop. Surg. Res..

[34] Lin M, Wen X, Huang Z (2023). A nomogram for predicting residual low back pain after percutaneous kyphoplasty in osteoporotic vertebral compression fractures. Osteoporos. Int..

[35] Bayram S, Akgül T, Adıyaman AE (2020). Effect of sarcopenia on mortality after percutaneous vertebral augmentation treatment for osteoporotic vertebral compression fractures in elderly patients: A retrospective cohort study[J]. World Neurosurg..

